# Double-positive T Lymphocytes Do Not Vary in Different Age Groups in Colombian Blood Donors

**DOI:** 10.4274/tjh.galenos.2020.2020.0017

**Published:** 2020-05-06

**Authors:** Miguel S. Gonzalez-Mancera, John Mario Gonzalez

**Affiliations:** 1 Universidad de los Andes, School of Medicine, Grupo de Ciencias Básicas Médicas, Bogotá, Colombia

**Keywords:** T lymphocytes, Flow cytometry

## To the Editor,

We read with interest the letter of Gonzalez-Mancera et al. [1] regarding the percentages and absolute numbers of double-positive T cells (DPTs) in the peripheral blood of a normal Italian population. In a previous article by our group, the DPT population was evaluated in one hundred suitable donors from a Colombian blood bank using flow cytometry. Our main findings showed a median DPT value of 2.6% and a higher percentage in women.

**Figure 1:**
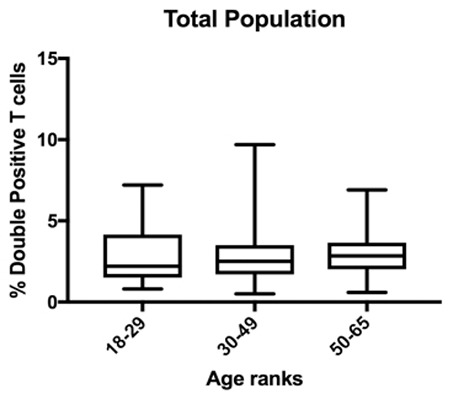
Median and interquartile rankings of donors according to age group. Kruskal-Wallis, p=0.83.

In the Italian cohort, they found an increase of DPTs with age and no difference by sex.  In our original study, we did not test donors over 61 years old to corroborate if age is associated with the marked increased level of DPTs above this age, as shown in the Italian population. We reanalyzed our data and did not find a difference in the percentages of DPTs when comparing age groups (Figure 1).

In the Spanish and German cohorts, although there was no significant difference in DPTs according to sex, women showed a tendency to have more DPTs when compared to men [2,3].

The flow cytometry panel (monoclonal antibodies and fluorochromes) used in our work detected and discriminated the DPTs through manual gating as shown in the original publication [1]. Previous studies showed that the antibody cocktail and the gating strategy (manual versus automated) are sources of variability in the results [4]. Also, according to our original flow cytometry analysis [1], it was possible to determine the subpopulations of CD4^high^CD8^low^ and CD4^low^CD8^high^ in healthy donors as described by other authors [5,6].

In order to understand the differences found in these publications, future studies must include a more diverse population, larger samples, and increased age range.
